# Preventing a loss of accuracy of the tennis serve under pressure

**DOI:** 10.1371/journal.pone.0255060

**Published:** 2021-07-26

**Authors:** Jürgen Beckmann, Lukas Fimpel, V. Vanessa Wergin

**Affiliations:** 1 Department of Sport and Health Sciences, Technical University of Munich, Munich, Germany; 2 School of Movement and Nutrition Science, University of Queensland, Brisbane, Australia; Universidade Federal de Mato Grosso do Sul, BRAZIL

## Abstract

Dynamically squeezing the left hand (left hand dynamic handgrip) has been shown to be effective in preventing choking under pressure in right-handers in a variety of sports. The current study assessed the effectiveness of the left hand dynamic handgrip in preventing a loss of accuracy of tennis serves in competitive situations. Twenty right-handed highly skilled junior athletes performed eight tennis serves at a target without pressure (pre-test), followed by eight serves under pressure (post-test). Ten of the participants conducted the left hand dynamic handgrip prior to the post-test, while the other ten performed an equivalent handgrip with their right hand. The serving accuracy of the group performing the handgrip with their right hand decreased significantly from pre- to post-test, while the accuracy of the left hand dynamic handgrip group remained stable. The results indicate the left hand dynamic handgrip to be effective in preventing reduced accuracy of the tennis serve in competition situations as a form of choking under pressure. This technique could easily be integrated into tennis players’ serving routines and promote stable match performance in competitions.

## Introduction

A tennis player has practiced their serve a thousand times until it has become completely automated, but when serving to win an important tournament, mistakes occur even in highly skilled players. Such a performance collapse has been referred to as choking under pressure, a metaphorical expression that describes performance decrements under pressure conditions despite an individual striving to perform well [1]. Pressure has been defined as any factor or combination of factors that increases the importance of optimal or superior performance and includes competition, the presence of an audience, reward or punishment contingency, and ego relevance [2]. In the present study, we investigated whether an embodiment technique, referred to as “dynamic handgrip” [3], could eliminate negative effects of the pressure experienced in a competitive situation on the tennis serve.

The concept of choking under pressure has been criticized as being vague regarding when performance failure should be called “choking” [4]. Certainly, a given performance can be labelled choking only if it is obvious that the individual had the intention to and yet was unable to perform better. Hill, Hanton, Fleming, and Matthews [5] discuss whether any underperformance should be labelled as choking and recommend using the term choking only for acute and significant underperformances that occur when the athlete perceives the demands of the situation to be higher than their coping capabilities. Based on this argument, Mesagno and Hill [6, p.272] propose a definition of choking as an acute and considerable decrease in skill execution and performance when self-expected standards are normally achievable, resulting from an increased anxiety under perceived pressure. With this definition, they emphasize the importance of pressure and anxiety in evoking choking.

Research shows that athletes experience choking under pressure in situations with high pressure because they either focus so much on the movement that they disturb the movement (e.g., [1,7,8]) or because they are distracted by task irrelevant thoughts (e.g., [9,10]). In this context, Mesagno and Beckmann [11] argue that anxiety is a major determinant for the occurrence of choking under pressure. Both attention-based models of high self-focus and distraction postulate that the anxiety to fail when it really counts is what leads to a shift of attention away from an optimal concentration on the task to an attentional focus on aspects that are not functional for performance (e.g., the audience). The anxiety to fail may also lead athletes to focus on internal aspects, such as details of skill execution or emotional reactions.

Neuroscience has identified neural correlates of this anxiety. Experts and elite athletes show a high automation of motor skills (e.g., [12]). In neuroscience, this is referred to as high neural efficiency because a limited number of neuronal connections in the brain is sufficient to generate the skill execution in contrast to a novice (e.g., [13,14]). Particularly, conscious control is dispensable for the execution of the skill. An athlete performing a tennis serve for example may try to perform especially well in a tournament and thus try to consciously control the countermovement, which disturbs the routinely movement of the serve. In fact, an attempt to consciously control the execution of a highly automated skill can interfere with a smooth execution, resulting in an increased kinematic variance [15]. The anxiety occurring in competitions can prompt a reinvestment of conscious attention on highly automated processes, which interferes with the performance of motor tasks [16]. EEG studies show a strong activation of brain areas related to conscious processing in experienced athletes who choke under pressure compared to athletes who do not choke. For example, in right-handed golfers, an increased interaction between the left temporal and frontal regions (i.e., temporal-frontal connectivity), which is associated with movement specific conscious processing, was found when they choked on a golf putting task [17]. Gallicchio, Cooke, and Ring [18] provided evidence that left temporal-frontal connectivity is an indicator of movement specific conscious processing in golfers. It is reduced in experts compared to novices and increased when an athlete fails on a motor task. Gallicchio, Cooke, and Ring [19] furthermore showed that practicing a motor task decreases conscious processing and improves performance.

Based on the assumptions about neurophysiological correlates of choking under pressure, a number of interventions have been developed to stabilize performance under pressure (see [11]). The present study focused on the problem of dominant activation of the left brain hemisphere associated with increased cognitive control [17]. Several studies in social psychology have used a clenching of the left hand to eliminate dominant left hemispheric activation (e.g., [20]). Beckmann, Gröpel, and Ehrlenspiel [3] tested the assumption that increasing activation in the right brain hemisphere through clenching the left hand would reduce left hemispheric domination and thereby eliminate choking under pressure. In three studies, results found that squeezing a ball with the left hand for 45 seconds eliminated choking under pressure in experienced right-handed athletes. The studies showed that a reduced accuracy in football penalty shooting, taekwondo kicks, and badminton serves under pressure was prevented through squeezing the ball with the left hand before executing the skill. In the control groups of all three studies, participants squeezed the ball with the right hand, which did not eliminate choking. However, EEG studies [21,22] showed that the hand clenching does not produce a shift of activation from the left to the right brain hemisphere but triggers relaxation (high alpha wave) that spreads across the whole cortex, producing a state of reduced cortical activity in the left brain hemisphere. In line with this, Hoskens et al. [23] argue that an engagement in movement control is associated with verbal-analytical processing (i.e., the planning and reasoning of an action framed in words). They further show that unilateral hand contractions influence the extent of this verbal-analytical engagement during motor planning, which in turn influences motor performance. The hand clenching could therefore be referred to as a reset mechanism. This effect occurs only with left hand clenching. Cross-Villasana, Gröpel, Doppelmayr, and Beckmann [21] in this context propose that the effectivity of left hand clenching compared to right hand clenching is due to higher levels of white matter and a higher connectivity of the right hemisphere (which is associated with the left hand), compared to the rest of the brain. This higher connectivity in the right brain hemisphere is assumed to lead to more reduction of cortical activity and evoke higher alpha waves when activated, compared to a similar activation of the left brain hemisphere [21]. Studies also suggest that a dynamic handgrip that meets a certain resistance (like that of a tennis ball) produces stronger effects than a static handgrip, because more power is needed to squeeze it, causing a higher activation in the corresponding brain hemisphere [21,24].

In addition to the above mentioned study by Beckmann, Gröpel, and Ehrlenspiel [3], a field study in artistic gymnastics was conducted [25]. The performance in the finals of the German University Championship was compared to the performance in the qualifications. Whereas in the previous studies right hand dynamic handgrip was used as the control condition, the control group in this field study did not engage in a hand clenching task. The time for the left hand dynamic handgrip was reduced to 10–15 seconds prior to the execution of the routine. Whereas the gymnasts not executing the left hand dynamic handgrip received worse evaluations in the finals compared to the qualifiers, the points assigned to those who used the left hand dynamic were not significantly reduced. Mesagno, Beckmann, Wergin, and Gröpel [26] applied the left hand dynamic handgrip in a study investigating the effects of different pre performance routines on the shooting accuracy of bowling players and found it to be effective in preventing choking under pressure as well. They had participants squeeze a ball twice per second for a duration of 10 seconds, which is a design that was also applied in a study by Wergin, Beckmann, Gröpel, and Mesagno [27] with beach volleyball players.

In many sports, the accuracy of the execution of a motor skill determines success or failure. In tennis, the serve plays an important role in winning a game. The faster the ball and the closer it is placed to the left or right corner of the service court, the higher the likelihood to score becomes, as the probability of the opponent player to return the ball diminishes [28]. A reduced accuracy of the serve in a tennis match compared to the standard serve accuracy of a player will decrease the player’s chances to win the match [29]. This could be referred to as a form of choking under pressure.

The current study aims to investigate whether similar effects of the left hand dynamic handgrip can be found in a realistic tennis service situation under pressure. The tennis serve is used as the motor skill as it is the only stroke that is performed without direct influence of the opponent and is therefore well suited as a standardized movement task. It is also one of the most important game situations [30,31], as it constitutes the beginning of every rally and thus has a direct impact on the further development of the rally. The server not only has a higher likelihood of winning points [32], a good serve offers the player the opportunity to force a quick and easy point win, while a bad serve gives the opponent the chance to attack and dominate the rally. This shows the special meaning and importance of a successful serve. Another reason why the tennis serve is an ideal task to examine effects of the left hand dynamic handgrip on performance under pressure is that it allows time for the opportunity to think, ruminate, and engage in self-talk and thus is predestined to activate the left brain hemisphere. Furthermore, it is hypothesized that participants performing the left hand dynamic handgrip will be less susceptible to choking under pressure and thus show fewer decrements in serving accuracy when exposed to pressure than participants performing the handgrip with their right hand.

## Materials and methods

### Participants

An a priori G*Power calculation [33] revealed that a sample size of 19 or more participants would provide sufficient power (.80) with significant effects at an alpha level of .05. A total of *N* = 20 male tennis players participated in the study. All participants were highly skilled junior athletes and played at least in the 4^th^ German league. The average age of the players was 17.45 years (*SD* = 0.51) ranging between 17 and 18 years. All players indicated right-hand dominance. Participants practiced on four to six days per week in one or two sessions per day on the tennis courts. Additionally, they participated in two athletic training sessions per week for 30–60 minutes focusing on strength and conditioning.

### Experimental setup and task

The experiment was conducted in a conventional tennis hall with three parallel clay courts. Compared to outdoor courts, the hall guarantees largely constant environmental conditions. The clay courts offer an easy way to measure serving accuracy due to the clearly visible impact point of the ball.

**Fig 1:**
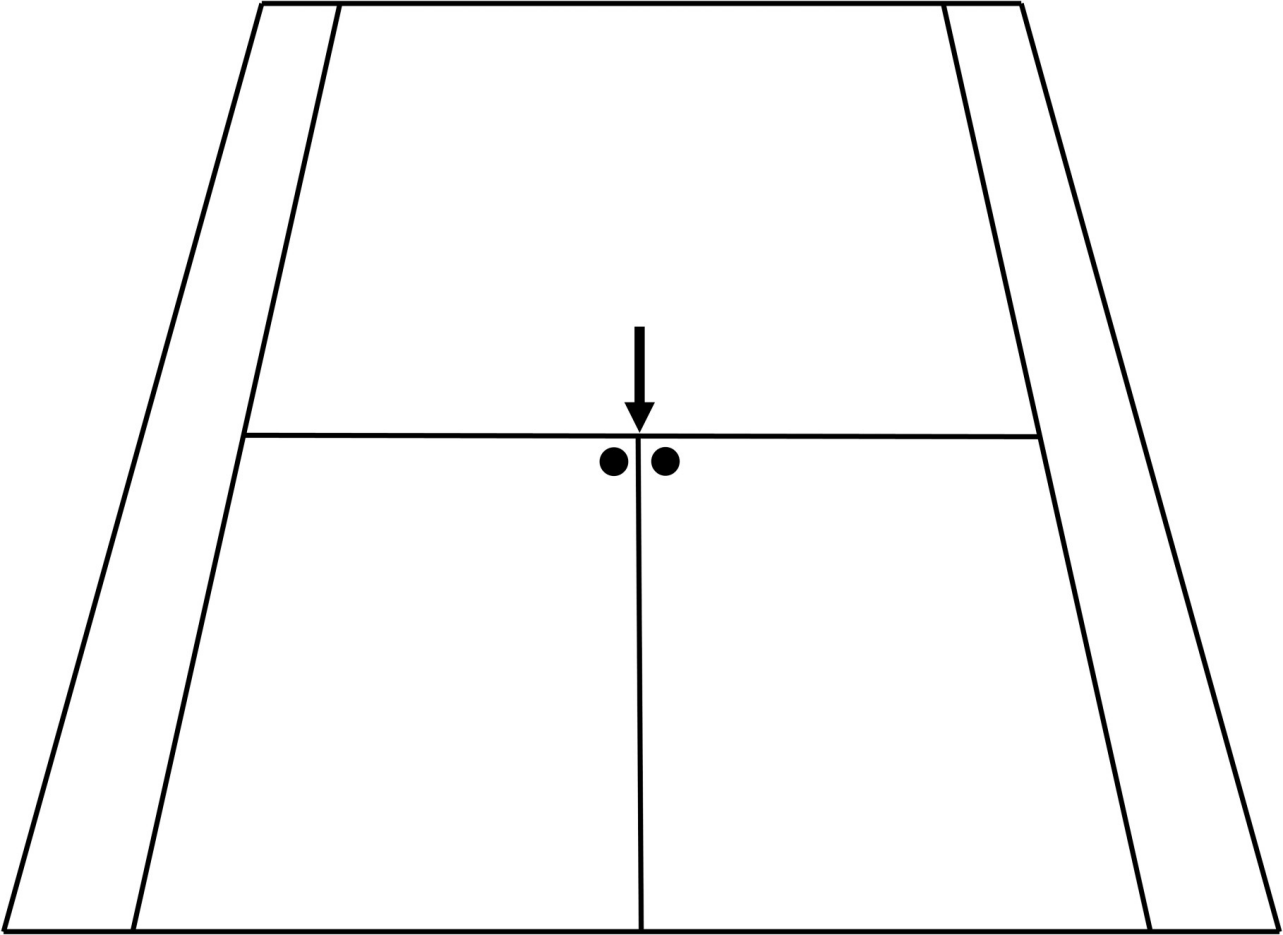
Illustration of target position for the serve. Target points for the serve at the opposite side of the court marked as black dots and starting point of the measurement marked with arrow. Left target point was served at from the right side of the baseline and right target point was served at from the left side of the baseline.

The participants’ task was to complete a series of eight serves in situations without pressure (pre-test) and another eight serves in situations under pressure (post-test). Players were not limited to a specific serving technique and instructed to serve with whatever style they preferred. The serves under pressure were conducted after participants performed either the left hand dynamic handgrip or a handgrip with their right hand. A series of eight serves was chosen because eight serves are the maximum number of serves performed by a player to impose a service game (i.e., to score every ball served and win the point) and reflect the average number of serves in a game [34]. The side was changed after every other serve, which corresponds to the practical situation in competitions, where the serve side is switched after every or every second service in case of a service fault. In addition, switching sides helps to compensate any player’s weaknesses or preferences for one side. Players took approximately 15 seconds between serves from the same side to prepare for the next serve and approximately 45 seconds to switch sides and prepare themselves for the serve in the pre-test. In the post-test, they took approximately 30 seconds to perform the handgrip and prepare themselves for the serve when serving from the same side and approximately 60 seconds to enter the court after their opponent, perform the handgrip, and prepare themselves for the serve when switching sides. The total duration of eight serves and measurements took approximately four minutes in the condition without pressure and approximately six minutes per player (12 minutes in total) in the condition under pressure. A target point for the serves was determined prior to the examination in cooperation with the coaches. Different tactical considerations result in different placement possibilities in competition. One of the most played variants is the straight service through the middle [30]. In this variant of the serve, which was chosen for the study, the ball should be placed as close as possible to the T-line and the inner line of the service field (Fig 1).

**Fig 2:**
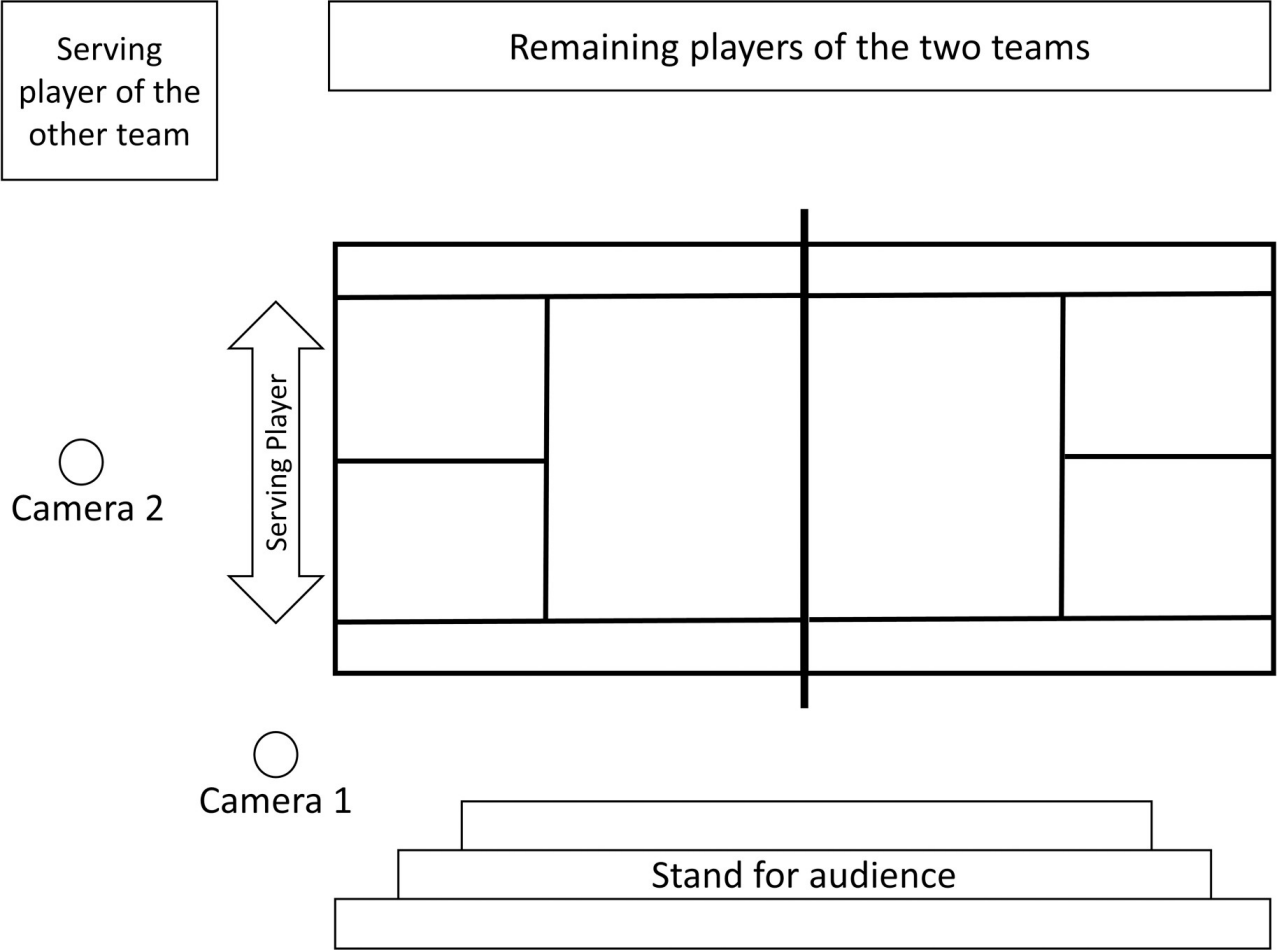
Experimental setup for the post-test phase performed under pressure.

The distance from the T-line to the nearest edge of the impact point of the ball was measured. Using a small pin, similar to a nail, anchored to the ground at the T-line and a tape measure attached to the pin, the exact distance from the T-line to the impact of the ball was determined in centimetres. This procedure is similar to accuracy measurements in other research investigating the effectiveness of pre performance routines (e.g., [26,27]). New official match balls of the Württemberg Tennis Association (WTB), the Dunlop Fort Tournament balls, were used in the study. All participants used their own rackets. Two video cameras were installed to generate pressure during the post-test phase. One camera was placed near to the baseline and the other was placed behind the player (Fig 2).

### Measures

Two questionnaires for the self-assessment of participants were used for data collection. First, a German version of the Edinburgh Handedness Inventory [35] was handed out to participants. The purpose of this questionnaire is to determine an individual’s handedness and its degree. It consists of 10 items related to everyday situations or movements (e.g., cutting, writing, brushing teeth) and with which hand they are preferably performed. The results can range between -100 (extremely left-handed person) and +100 (extremely right-handed person). Good internal consistency and validity of the test were shown in previous studies (e.g., [36]). In this study, a participant with a value of at least +50 was regarded as right-handed [35].

Second, a German version (WAI-S, [37]) of the Competitive State Anxiety Inventory [38] was used to assess cognitive and somatic anxiety in participants and check, whether the pressure induction was successful. The questionnaire contains 12 items on current emotions and perceived somatic signs. Answers to each item are given on a four-point Likert scale ranging from 1 = “not at all” to 4 = “very true”. The test retrieves three different components of competition anxiety: somatic anxiety, cognitive anxiety, and confidence. In the current study, only the eight items of the cognitive and somatic anxiety subscales were used, as they assess the main aspects of anxiety. Research indicates that the WAI-S shows sufficient internal consistency and validity if applied as a state measure in performance-related settings (e.g., [37,39]).

### Experimental groups

Participants were randomly assigned to one of two groups of equal size. Both groups performed the same task in the condition without pressure but varied in their tasks in the pressure condition. The first group, subsequently referred to as “right hand group”, was instructed to squeeze their racket grip with the right hand twice a second for 10–15 seconds with submaximal strength immediately before each serve performed under pressure, as 10–15 seconds of squeezing have been shown to be effective in gymnasts [25] and bowling players [26]. Participants of the second group, hereafter referred to as “left hand group”, performed the left hand dynamic handgrip by dynamically squeezing the tennis ball with their left hand for 10–15 seconds immediately before each serve in the pressure condition. The tennis ball or the racket grip were chosen as objects to be squeezed by the players to allow for maximal practical applicability of the intervention task. The time span between the performed handgrip and the serve was approximately 0–5 seconds.

The right hand group performed a similar task to the left hand group with the other hand, in order to create equal conditions. Furthermore, recent studies [40] indicate that small behavioral steps like this can increase concentration and performance in competition.

### Pressure manipulation

Several steps were taken to induce pressure during the post-test. The post-test itself was designed as a competition between the two groups. The average distance to the T-line of the serves of all players of a team were added up to a team score. The group with the smaller deviation score won the competition. Two competing players, one of the right hand and one of the left hand group, performed a series of two practice and eight measured serves alternately in a direct comparative and competitive situation. After every other serve, the distance was measured and announced loudly. Thus, the teams received direct feedback about their intermediate results, which was intended to further increase pressure. As an additional incentive to perform well, sweatbands, water bottles, and energy bars were provided as a prize for the winning team. In order to simulate actual competition and to increase pressure further, 28 spectators (unknown to the players) were present during the post-test and two video cameras were set up. Players were informed that a video analysis of the technical execution of their movement would be performed later on.

### Procedure

The requirement to obtain full ethical approval for the study was waived, as the study was conducted in line with the guidelines of the German Research Foundation (DFG) and the Department of Sport and Health Science at the Technical University of Munich. Participation in the study was voluntary and all participants gave written informed consent in accordance with the Declaration of Helsinki. Furthermore, participants were able to withdraw from the research at any time without consequence. The study did not involve any invasive or potentially dangerous methods and the setup did not mine the safety of participants. Data were anonymized completely and appropriate procedures for confidentiality were applied. Players were recruited through a German tennis club and coaches working in the club. When they agreed to participate, they were invited to a meeting with coaches and the experimenter prior to data collection. During this meeting, the procedure of the investigation was described and open questions of participants were answered. The true background of the investigation was not revealed to players at this time. Instead, they were told that they would participate in a study on the importance of concentration in tennis. They were further informed that a simple motor task (hand contraction) prior to the movement task could increase concentration and it should be investigated whether an increased concentration would affect their serving accuracy. Informed consent in accordance with the Declaration of Helsinki was obtained from all participants.

Upon arrival at the tennis hall, participants filled out the Edinburgh Handedness Inventory [35]. Afterwards a tennis coach conducted a warm-up. Subsequently, all participants completed a five-minute stroke session including baseline shots, volleys, as well as serves. At the beginning of the pre-test phase, after the participants were instructed and just before they started with their two practice serves, participants completed the WAI-S [37] for the first time as a baseline anxiety measure. During the pre-test, each player first completed four test serves, which were not measured, two from the right and two from the left side of the baseline. Participants then performed eight measured serves. The task of the players was to serve the ball through the middle aiming at the defined target point (Fig 1). The distance was measured after two serves and the court was swept to avoid confusion of several impact points. While all serves were registered, only valid serves (i.e., serves hit inside the service area) were used for statistical analyses. This was the more conservative measure, as some serves were placed closely to the target but still hit “out”, which is why they would not have counted in a tournament as well. During a player’s service series, the remaining players stayed on the side courts to ensure they were not in view of the player being tested and to assure there was no distraction or potential pressure exerted through observation by others. Once all players had finished their serves in the pre-test condition, there was a 30-minute break before moving on to the post-test.

Both groups received a short instruction on the handgrip tasks prior to the post-test. After the introduction of the handgrip task, participants were informed about the competition conditions and the prizes for the winning team. Once participants were informed and had no more questions, (just before the start of the competition), the players filled out the WAI-S [37] for a second time to examine their anxiety levels in the pressure situation of the post-test. After the successful execution of the competition, the winning team received the prize and all participants were informed about the actual background of the investigation.

## Results

### Homogeneity of groups

An independent t-test comparing pre-test serving accuracy between the groups revealed no significant difference in relation to serving accuracy of valid serves between right hand (*M* = 27.10, *SD* = 8.42) and left hand group (*M* = 29.74, *SD* = 8.40), *t*(18) = -.71, *p* = .492, *d* = 31, 95% CI = [-10.54, 5.26]. Therefore, we assumed equal group-based serving accuracy.

### Experienced pressure (state anxiety)

A 2 x 2 (Group x Phase) repeated measures Multivariate Analyses of Variance (MANOVA) indicated a main effect of Phase on cognitive and somatic anxiety (*F*(2, 17) = 88.26, *p* < .001, ηp^2^ = .91), but no main effect of Group on cognitive and somatic anxiety (*F*(2, 17) = 1.55, *p* = .241, ηp^2^ = .15). No interaction was found between Group and Phase (*F*(2, 17) = 1.07, *p* = .364, ηp^2^ = .11). Both cognitive (*F*(2, 17) = 131.72, *p* < .001, ηp^2^ = .88) and somatic anxiety scores (*F*(2, 17) = 91.41, *p* < .001, ηp^2^ = .84) increased from pre- to post-test but independent of group belongingness (see Table 1 for means and standard deviations). Thus, the pressure induction was considered successful in both groups.

**Table 1:** Means and standard deviations for cognitive anxiety, somatic anxiety, and accuracy of valid serves.

Group	Cognitive Anxiety		Somatic Anxiety		Accuracy (MAD)	
	Pre-test	Post-test	Pre-test	Post-test	Pre-test	Post-test
LH group	7.00 (1.63)	11.80 (2.15)	5.60 (1.65)	11.20 (1.87)	29.74 (8.40)	29.74 (7.32)
RH group	5.70 (1.77)	11.00 (1.70)	6.50 (1.35)	10.80 (2.49)	27.10 (8.24)	34.39 (10.87)

Means and Standard Deviations in parentheses are shown in centimetres for both groups pre- and post-test. MAD = Mean Absolute Distance; LH = left hand; RH = right hand.

### Service accuracy

**Fig 3:**
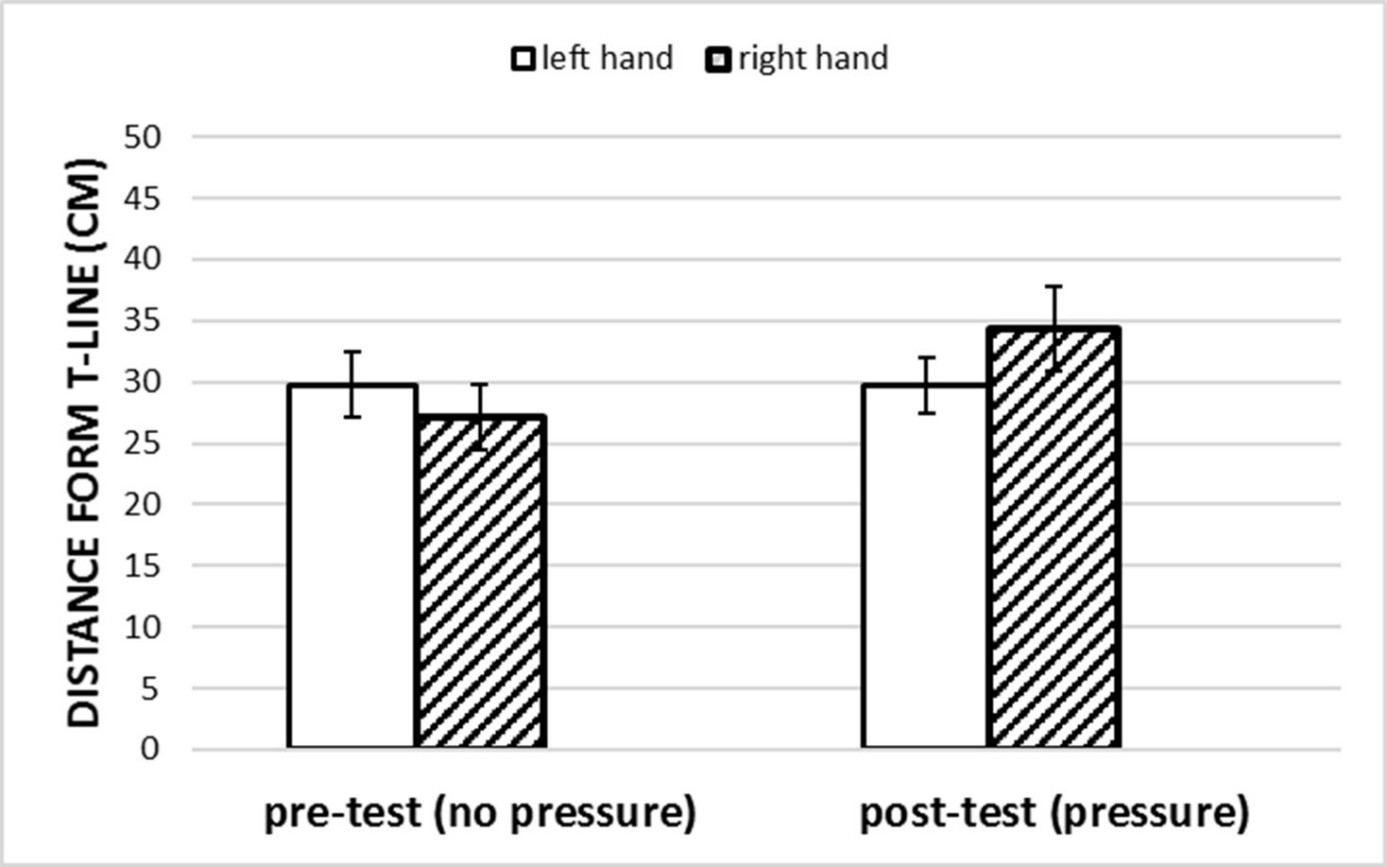
Interaction of phase and group on mean distance of valid serves from target of both groups pre- and post-test. Error bars represent standard errors.

A 2 x 2 (Group x Phase) repeated measures Analysis of Variance (ANOVA) revealed a main effect of Phase on service accuracy, (*F*(1, 18) = 11.90, *p* = .003, ηp^2^ = .40), indicating that the distance of valid serves from the target increased significantly from pre-test to post-test. Furthermore, an interaction of Phase and Group on service accuracy was found (*F*(1, 18) = 11.88, *p* = .003, ηp^2^ = .40). The distance of valid serves from the target of the right hand group increased from pre-test to post-test, indicating a decrease in performance, while service accuracy of valid serves of the left hand group remained stable (Fig 3). Means and standard deviations of the service accuracy can be found in Table 1.

## Discussion

The present study investigated the effectiveness of the left hand dynamic handgrip as an intervention strategy to prevent reduced accuracy of the tennis serve under pressure experienced in a simulated match situation. The left hand dynamic handgrip was integrated into the regular preparation routine for the serve, while the duration of its performance was reduced to 10–15 seconds, similar to earlier studies [25,26], to make it more applicable within the routine.

In accordance with the hypothesis, tennis players utilising the left hand dynamic handgrip were able to serve just as accurate under pressure as they did under no pressure, while serving accuracy of participants performing a right hand dynamic handgrip worsened significantly under pressure, indicating a worsening of performance. This main finding provides additional support for the alleviating effect of the left hand dynamic handgrip on choking under pressure [3,25,26].

Compared to the previous studies, participants of the current study performed the dynamic handgrip for a relatively short amount of time (10–15 seconds), which appeared to be sufficient in reducing negative effects of choking. This finding constitutes an important practical implication, as it demonstrates the applicability of the left hand dynamic handgrip in actual tennis competitions. It could easily be performed as a strategy in preparation of the next serve in a regular tennis match. The left hand dynamic handgrip could become part of a serving routine, which players usually perform prior to serving (e.g., [41]). In case studies Gröpel, Mesagno, and Beckmann [42] found that athletes accepted to integrate the handgrip into their routines and did not need much time to get accustomed with this relatively simple embodiment technique.

The current study therefore supports the efficacy of a realistic prevention strategy for choking under pressure in tennis players. In line with findings of Cross-Villasana et al. [21], it is assumed that performing the left hand dynamic handgrip leads to an increase in alpha in the whole cortex and a relaxation of the cortex as a consequence. This increase in alpha likely alleviates an increased activity in the left hemisphere linked to choking under pressure. Accordingly, performing the left hand dynamic handgrip may help tennis players avoid any verbal-analytical processing (e.g., related to negative thoughts) or conscious processing (e.g., related to a conscious control of movements) under pressure and thus maintain their level of performance.

In the present study, athletes performed eight serves without pressure and eight further serves in a condition under pressure. In a realistic game situation, it is likely that even further increased perceived pressure could be observed between first and second serves. When the first service is a fault, pressure on players increases and they might tend to use a “play it safe” strategy for the second service in order to prohibit another fault [31]. Therefore, it would be interesting to investigate the effectiveness of the left hand dynamic handgrip in relation to the second service. Future studies could make the competitive situation more realistic by using a first and a second service to investigate whether faults in the first serve increase pressure on the second service and whether this pressure could be reduced by the intervention. The “play it safe” strategy could also involve a reduced force of the serve, as players may play less risky to make sure the ball is placed in the service court. Future studies could include an assessment of the ball speed to detect differences in this performance aspect.

However, the “play it safe” strategy regarding the tennis serve would not qualify as choking under pressure according to Hill et al. [5] and Mesagno and Hill [6], who state that choking should be associated with an acute and considerable decrease in skill execution rather than being used as a term for all deteriorations of performance. In fact, the “play it safe” strategy may avoid dramatic faults or double faults. Nevertheless, players do not play up to their potential because of an anxiety to fail caused by competitive pressure [11], thereby reducing their chances of winning. We are thus inclined to see this as choking under pressure and endorse further investigation.

### Limitations and future research

The study has some limitations. First, the sample size of 20 participants is rather small and a bigger sample may have revealed more or stronger effects between the two groups. Second, exclusively male junior tennis players participated in the study, which is why transferability of results among gender and age are limited. Third, as the sample consisted of right-handed players, the handgrip of the right hand group may have increased fatigue in players, which may have impacted serving accuracy. Fourth, serving accuracy was used as the only measure of performance in a relatively small number of trials. Thus, future studies should aim to investigate a higher number of athletes consisting of male as well as female players of different ages, considering other measures of performance, such as service velocity. Additionally, video recordings of athletes’ performance could be used in future studies to analyze visible changes in athletes’ behaviors and identify movement patterns related to the experience of choking under pressure [43]. As explained in more detail above, in tennis, an investigation of the impact of the left hand dynamic handgrip on first vs. second service would be of interest. Furthermore, future research should investigate the effectiveness of the left hand dynamic handgrip in various tasks of other sports. As it appears to be working well in accuracy tasks like the tennis serve or bowling shots [26], this technique could be employed in any other racket sport, such as badminton or table tennis, as well as in sports related to shooting (e.g., archery, modern pentathlon, military pentathlon, biathlon). While this study provides support for the effectiveness of the left hand dynamic handgrip in preventing a loss of accuracy in accuracy tasks under pressure, questions about its execution remain. Thus, future studies should also focus on investigating the ideal duration of execution of the left hand dynamic handgrip as well as the duration of its effect on performance.

### Conclusions

The study provides further support for the assumption that the left hand dynamic handgrip may be a useful tool in countering performance decrements evoked by competitive pressure. Although the underlying mechanisms of the left hand dynamic handgrip require further clarification, it has been shown to be an effective prevention strategy to prohibit choking under pressure in tennis serving, which can easily be implemented in athletes’ existing serving routines. Future research should investigate the applicability of the left hand dynamic handgrip in other sports and sports tasks related to accuracy.
